# Stratifying risk of Alzheimer’s disease in healthy middle-aged individuals with machine learning

**DOI:** 10.1093/braincomms/fcaf121

**Published:** 2025-03-25

**Authors:** Raghav Tandon, Liping Zhao, Caroline M Watson, Neel Sarkar, Morgan Elmor, Craig Heilman, Katherine Sanders, Chadwick M Hales, Huiying Yang, David W Loring, Felicia C Goldstein, John J Hanfelt, Duc M Duong, Erik C B Johnson, Aliza P Wingo, Thomas S Wingo, Blaine R Roberts, Nicholas T Seyfried, Allan I Levey, James J Lah, Cassie S Mitchell

**Affiliations:** Department of Biomedical Engineering, Georgia Institute of Technology, Atlanta, GA 30332, USA; Center for Machine Learning, Georgia Institute of Technology, Atlanta, GA 30332, USA; Department of Biostatistics and Bioinformatics, Emory School of Public Health, Atlanta, GA 30322, USA; Emory Goizueta Alzheimer’s Disease Research Center, Atlanta, GA 30329, USA; Emory Goizueta Alzheimer’s Disease Research Center, Atlanta, GA 30329, USA; Department of Neurology, Emory School of Medicine, Atlanta, GA 30322, USA; Department of Biomedical Engineering, Georgia Institute of Technology, Atlanta, GA 30332, USA; Emory Goizueta Alzheimer’s Disease Research Center, Atlanta, GA 30329, USA; Department of Neurology, Emory School of Medicine, Atlanta, GA 30322, USA; Emory Goizueta Alzheimer’s Disease Research Center, Atlanta, GA 30329, USA; Department of Neurology, Emory School of Medicine, Atlanta, GA 30322, USA; Emory Goizueta Alzheimer’s Disease Research Center, Atlanta, GA 30329, USA; Department of Neurology, Emory School of Medicine, Atlanta, GA 30322, USA; Emory Goizueta Alzheimer’s Disease Research Center, Atlanta, GA 30329, USA; Department of Neurology, Emory School of Medicine, Atlanta, GA 30322, USA; Center for Neurodegenerative Disease, Emory University, Atlanta, GA 30322, USA; Department of Biostatistics and Bioinformatics, Emory School of Public Health, Atlanta, GA 30322, USA; Emory Goizueta Alzheimer’s Disease Research Center, Atlanta, GA 30329, USA; Emory Goizueta Alzheimer’s Disease Research Center, Atlanta, GA 30329, USA; Department of Neurology, Emory School of Medicine, Atlanta, GA 30322, USA; Emory Goizueta Alzheimer’s Disease Research Center, Atlanta, GA 30329, USA; Department of Neurology, Emory School of Medicine, Atlanta, GA 30322, USA; Department of Biostatistics and Bioinformatics, Emory School of Public Health, Atlanta, GA 30322, USA; Emory Goizueta Alzheimer’s Disease Research Center, Atlanta, GA 30329, USA; Emory Goizueta Alzheimer’s Disease Research Center, Atlanta, GA 30329, USA; Department of Neurology, Emory School of Medicine, Atlanta, GA 30322, USA; Department of Biochemistry, Emory School of Medicine, Atlanta, GA 30322, USA; Emory Goizueta Alzheimer’s Disease Research Center, Atlanta, GA 30329, USA; Department of Neurology, Emory School of Medicine, Atlanta, GA 30322, USA; Center for Neurodegenerative Disease, Emory University, Atlanta, GA 30322, USA; Department of Psychiatry, Emory School of Medicine, Atlanta, GA 30322, USA; Division of Mental Health, Atlanta VA Medical Center, Atlanta, GA 30033, USA; Emory Goizueta Alzheimer’s Disease Research Center, Atlanta, GA 30329, USA; Department of Neurology, Emory School of Medicine, Atlanta, GA 30322, USA; Center for Neurodegenerative Disease, Emory University, Atlanta, GA 30322, USA; Center for Neurodegenerative Disease, Emory University, Atlanta, GA 30322, USA; Department of Biochemistry, Emory School of Medicine, Atlanta, GA 30322, USA; Emory Goizueta Alzheimer’s Disease Research Center, Atlanta, GA 30329, USA; Center for Neurodegenerative Disease, Emory University, Atlanta, GA 30322, USA; Department of Biochemistry, Emory School of Medicine, Atlanta, GA 30322, USA; Emory Goizueta Alzheimer’s Disease Research Center, Atlanta, GA 30329, USA; Department of Neurology, Emory School of Medicine, Atlanta, GA 30322, USA; Center for Neurodegenerative Disease, Emory University, Atlanta, GA 30322, USA; Emory Goizueta Alzheimer’s Disease Research Center, Atlanta, GA 30329, USA; Department of Neurology, Emory School of Medicine, Atlanta, GA 30322, USA; Center for Neurodegenerative Disease, Emory University, Atlanta, GA 30322, USA; Department of Biomedical Engineering, Georgia Institute of Technology, Atlanta, GA 30332, USA; Center for Machine Learning, Georgia Institute of Technology, Atlanta, GA 30332, USA

**Keywords:** asymptomatic Alzheimer’s disease, CSF proteomics, disease progression, machine learning, risk stratification

## Abstract

Alzheimer’s disease has a prolonged asymptomatic phase during which pathological changes accumulate before clinical symptoms emerge. This study aimed to stratify the risk of clinical disease to inform future disease-modifying treatments. Cerebrospinal fluid analysis from participants in the Emory Healthy Brain Study was used to classify individuals based on amyloid beta 42 (Aβ42), total tau (tTau) and phosphorylated tau (pTau) levels. Cognitively normal (CN), biomarker-positive (CN)/BM+individuals were identified using a tTau: Aβ42 ratio > 0.24, determined by Gaussian mixture models. CN/BM+ individuals (*n* = 134) were classified as having asymptomatic Alzheimer’s disease (AsymAD), while CN, biomarker-negative (CN/BM−) individuals served as controls (*n* = 134). Cognitively symptomatic, biomarker-positive individuals with an Alzheimer’s disease diagnosis confirmed by the Emory Cognitive Neurology Clinic were labelled as Alzheimer’s disease (*n* = 134). Study groups were matched for age, sex, race and education. Cerebrospinal fluid samples from these matched Emory Healthy Brain Study groups were analysed using targeted proteomics via selected reaction monitoring mass spectrometry. The targeted cerebrospinal fluid panel included 75 peptides from 58 unique proteins. Machine learning approaches identified a subset of eight peptides (ADQDTIR, AQALEQAK, ELQAAQAR, EPVAGDAVPGPK, IASNTQSR, LGADMEDVCGR, VVSSIEQK, YDNSLK) that distinguished between CN/BM− and symptomatic Alzheimer’s disease samples with a binary classifier area under the curve performance of 0.98. Using these eight peptides, Emory Healthy Brain Study AsymAD cases were further stratified into ‘Control-like’ and ‘Alzheimer’s disease-like’ subgroups, representing varying levels of risk for developing clinical disease. The eight peptides were evaluated in an independent dataset from the Alzheimer’s Disease Neuroimaging Initiative, effectively distinguishing CN/BM− from symptomatic Alzheimer’s disease cases (area under the curve = 0.89) and stratifying AsymAD individuals into control-like and Alzheimer’s disease-like subgroups (area under the curve = 0.89). In the absence of matched longitudinal data, an established cross-sectional event-based disease progression model was employed to assess the generalizability of these peptides for risk stratification. In summary, results from two independent modelling methods and datasets demonstrate that the identified eight peptides effectively stratify the risk of progression from asymptomatic to symptomatic Alzheimer’s disease.

## Teaser

Tandon *et al*. report that machine learning analysis of cerebrospinal fluid proteins identifies eight peptides that discriminate between healthy controls and Alzheimer’s disease patients. These peptides effectively stratify cognitively normal individuals with underlying pathology into subgroups showing concordance with disease progression markers across two independent cohorts.

## Graphical Abstract

**Graphical Abstract:**
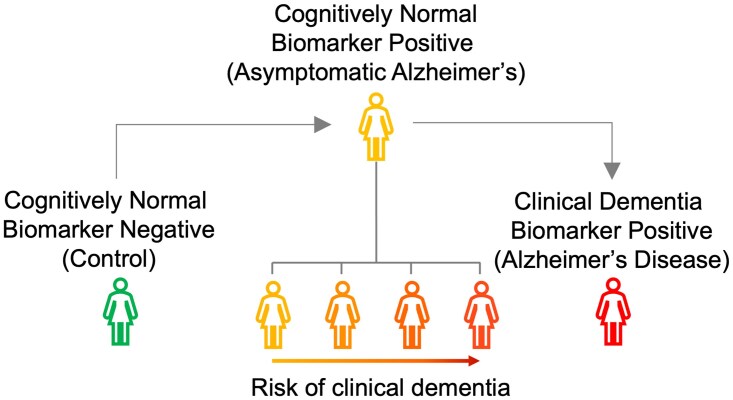

## Introduction

Pathophysiological changes of Alzheimer’s disease begin many years before the functional or cognitive decline associated with disease. The presence of pathology can be ascertained through cerebrospinal fluid (CSF) tests and positron-emission tomography (PET) scans. In individuals with dominantly inherited Alzheimer’s disease, CSF Tau begins to increase 15 years before symptom onset, while Aβ42 begins to decline over 20 years prior to symptom onset.^1,2^ Until a recent report of lecanemab,^3^ clinical trials of anti-amyloid monoclonal antibodies,^4-6^ secretase inhibitors,^7,8^ and anti-tau monoclonal antibodies^9,10^ have had limited success for disease modification in patients with symptomatic Alzheimer’s disease. Given the long evolution of these pathologies before clinical symptoms, identifying and treating at-risk individuals during asymptomatic stages may be a more effective strategy to delay or prevent dementia onset.^11^ Thus, a key to successful implementation of secondary prevention trials may lie in the ability to identify those at the greatest risk for Alzheimer’s disease prior to symptom onset. It is also important to recognize that many cognitively normal (CN) individuals may have evidence of Alzheimer’s disease neuropathology at death.^12,13^ This consideration is reinforced by examination of autopsy results available from the National Alzheimer’s Coordinating Center (data received May 2022). Among 787 individuals who donated their brains and were classified as normal controls at their last evaluation, 227 (28.8%) had moderate or frequent amyloid plaques (CERAD ≥2), 386 (49.0%) had neocortical neurofibrillary tangles (Braak ≥3) and 164 (20.8%) had both CERAD ≥2 and Braak ≥3 (J. Lah, unpublished). Therefore, simply identifying the presence of Alzheimer’s disease pathology does not imply a need for intervention.

For effective deployment of preventative therapies, it is imperative to both identify the presence of silent pathology and determine those at the greatest risk of developing symptomatic disease. Alzheimer’s disease is a multifactorial neurodegenerative disorder with numerous aetiopathogenic mechanisms. Thus, several factors may influence early disease evolution, including genetics, lifetime exposures and medical comorbidities. Additionally, Alzheimer’s disease typically manifests as mixed pathologies, which evolve and change over time.^14-16^ While biomarkers of amyloid plaques and neurofibrillary tangles provide high diagnostic accuracy for presence of disease pathology, multiple biomarkers are likely to be required to predict other underlying pathologies, disease stage and risk of clinical progression.

The complexity of Alzheimer’s disease pathophysiology necessitates comprehensive molecular profiling approaches. Recent proteomic studies have demonstrated the power of this strategy.^17-21^ Application of these systems biology approaches has led to development of proteomics-based CSF biomarker panels that link to distinct Alzheimer’s disease pathophysiological processes and differential expression in the CSF and brain, offering potential for proteomics-based Alzheimer’s disease biomarkers.^17^ Mass spectrometry-based analysis of >2000 brains and 400 CSF samples identified key protein modules linked to sugar metabolism, Alzheimer’s disease genetic risk factors and glial markers that correlate strongly with disease pathology and cognitive decline.^20^ Furthermore, targeted proteomic approaches have successfully identified CSF proteins that can distinguish both AT status and cognitive impairment, complementing traditional Aβ and Tau biomarkers.^21^ This work indicates that additional proteomics based biomarkers may have the ability to stratify risk of clinically asymptomatic Alzheimer’s disease.

To better understand the evolution of Alzheimer’s disease in its earliest stages, CSF characteristics were explored in a subset of CN middle-aged individuals (50–75 years) in the Emory Healthy Brain Study (EHBS^22^), including a subset of 134 individuals with CSF levels of Aβ42, total Tau (tTau) and phospho181-Tau (pTau) indicative of underlying Alzheimer’s disease pathology. This group of asymptomatic Alzheimer’s disease (AsymAD) individuals was demographically matched with groups of CN biomarker-negative (CN/BM−) controls and patients with biomarker-confirmed Alzheimer’s disease. In the EHBS cohort, CSF samples were examined by selected reaction monitoring mass spectrometry for levels of 75 putative Alzheimer’s disease biomarkers.^17^ Machine learning algorithms were used to identify a set of CSF peptides that effectively discriminate CN/BM− controls from symptomatic Alzheimer’s disease cases and sub-categorized AsymAD individuals into ‘Control-like’ and ‘Alzheimer’s disease-like’ groups. The discriminative peptide set was independently examined in a second data set from the Alzheimer’s Disease Neuroimaging Initiative (ADNI), which labelled subjects using AV45 and Fluorodeoxyglucose Positron Emission Tomography (FDG PET). Our results identify a set of CSF biomarkers that stratify risk of conversion from asymptomatic to symptomatic stages of Alzheimer’s disease.

## Materials and methods

### Emory health brain study

The EHBS^22^ is a longitudinal cohort study of CN adults (50–75 years) established in 2016. All participants provided consent according to the Declaration of Helsinki, and the study protocol was approved by the Internal Review Board of Emory University.

EHBS is a research study specifically focused on discovering biomarkers that predict Alzheimer’s disease and other dementias. EHBS participants are self-reported cognitively and functionally intact and free of pre-existing diagnosis of mild cognitive impairment (MCI) or any dementia. All participants complete biennial study visits which include neuropsychological testing, cardiovascular measures, brain imaging and biospecimen collection (blood, CSF). From this cohort, 134 CN, biomarker-positive (CN/BM+) individuals were identified with AsymAD based on measurements of Aβ42, tTau and pTau using a locally defined cut-off value for tTau:Aβ42 ratio (>0.24) identified by Gaussian mixture models.^23^ These individuals were matched for age, sex and race with 134 biomarker-negative CN/BM− controls and 134 patients with biomarker-confirmed symptomatic Alzheimer’s disease seen in the Emory Cognitive Neurology Clinic. AsymAD and CN/BM− controls were additionally matched for education. All individuals included in our analyses provided informed consent to participate in research protocols approved by the Emory University Institutional Review Board. Table 1 shows the descriptive statistics for the full EHBS cohort (*N* = 1149) and matched groups of clinical Alzheimer’s disease, AsymAD and CN/BM− groups (*N* = 134 each). Statistical differences between the AsymAD and CN/BM− groups were evaluated using the McNemar-Bowker’s test for categorical variables and by paired *t*-test or Wilcoxon signed rank test for continuous variables depending on the distribution.

**Table 1:** Patients characteristics

Characteristics	All EHBS (*N* = 1149)	Clinical Alzheimer’s disease (*N* = 134)	AsymAD (*N* = 134)	CN/BM− (*N* = 134)	*P-*value
Age, mean ± SD	62.7 ± 6.7	66.0 ± 5.8	66.0 ± 5.8	65.9 ± 6.0	0.11
Female, *n* (%)	797 (69.4)	100 (74.6)	100 (74.6)	100 (74.6)	0.99
Race, *n* (%)					0.99
Caucasian	1003 (87.3)	124 (92.5)	124 (92.5)	124 (92.5)	
African-American	125 (10.9)	9 (6.7)	9 (6.7)	9 (6.7)	
Asian	14 (1.2)	1 (0.7)	1 (0.7)	1 (0.7)	
Education, mean ± SD	16.7 ± 2.1	15.4 ± 2.6	16.7 ± 2.0	16.8 ± 2.3	0.69
MoCA, mean ± SD	26.6 ± 2.3	17.4 ± 5.5	26.3 ± 2.6	26.8 ± 2.0	0.07
*APOE* ε4 allele frequency	0.17	0.50	0.40	0.08	**<0**.**0001**
CSF analytes, median (IQR)					
Aβ_42_ pg/mL	1212.0 (894.9–1586.0)	540.7 (445.6–660.2)	740.1 (609.8–862.5)	1412.0 (1192–1700)	**<0**.**0001**
tTau pg/mL	174.2 (139.8–220.1)	343.2 (265.7–458.5)	242.0 (194.9–299.4)	167.6 (139.9–192.7)	**<0**.**0001**
pTau pg/mL	15.2 (12.0–19.7)	33.9 (26.7–47.3)	22.8 (18.6–28.2)	14.8 (12.2–17.2)	**<0**.**0001**
tTau:Aβ42 ratio	0.14 (0.12–0.18)	0.64 (0.49–0.86)	0.31 (0.27–0.42)	0.12 (0.11–0.13)	**<0**.**0001**
pTau:Aβ42 ratio	0.012 (0.011–0.016)	0.065 (0.047–0.088)	0.029 (0.025–0.042)	0.011 (0.002–0.011)	**<0**.**0001**

The table shows demographic features, cerebrospinal fluid (CSF) analytes levels for all Emory Healthy Brain Study (EHBS) subjects, including symptomatic Alzheimer’s Disease, asymptomatic Alzheimer’s Disease (AsymAD) that were cognitively normal biomarker positive (CN/BM+), and control subjects that were cognitively normal biomarker negative (CN/BM−). AsymAD cases and CN/BM− controls are matched for age, sex, race and education. *P-*values are for comparisons of AsymAD and CN/BM− groups. Continuous variables were compared by paired *t*-test or Wilcoxon signed rank test depending on the distribution. Categorical variables were compared with McNemar–Bowker’s test. Bolded *P*-values indicate statistical significance.

CSF samples from all participants were collected in a standardized fashion applying common preanalytical methods. EHBS participants were asked to fast for at least 6 h prior to study visits. Patients donating CSF samples during clinical evaluations were asked to fast prior to their lumbar puncture procedure, but failure to do so did not preclude lumbar puncture and CSF collection. Most, but not all procedures, were conducted before noon. All clinicians performing lumbar punctures in the Cognitive Neurology Clinic are also active investigators in the EHBS and apply shared standard work in both settings. Lumbar punctures are performed using a 24 g atraumatic Sprotte spinal needle (Pajunk Medical Systems, Norcross, GA, USA) with aspiration and, after clearing any blood contamination, CSF is transferred from syringe to 15 mL polypropylene tubes (Corning, Glendale, AZ, USA), which are inverted several times. The CSF is aliquoted without further handling into 0.5 mL volume in 0.9 mL FluidX tubes (Azenta, Chemsford, MA, USA) and placed into dry ice/methanol bath prior to transfer to −80°C freezers. Time from initial collection to storage at −80°C is <60 min. Aβ42, tTau and pTau assays were performed on CSF samples following a single freeze-thaw cycle on a Roche Cobas e601 analyzer using the Elecsys assay platform.^24^ All assays were performed in a single laboratory in the Emory Goizueta Alzheimer’s Clinical Research Unit following manufacturer’s recommended protocols, including daily QC samples for Aβ42, tTau and pTau to ensure reads within specified parameters. Locally generated bridging samples were included with new reagent lots to monitor for any drift.

### Alzheimer’s disease neuroimaging initiative

The ADNI was launched in 2003 as a public-private partnership, led by Principal Investigator Michael W. Weiner, MD. All ADNI participants provided consent according to the Declaration of Helsinki. ADNI study protocols were approved by the Institutional Review Boards of all participating institutions. Informed consent was obtained from all participants or their authorized representatives at each site.

The primary goal of ADNI has been to test whether serial magnetic resonance imaging, PET, other biological markers and clinical and neuropsychological assessment can be combined to measure the progression of MCI and early Alzheimer’s disease. Recent previously performed targeted proteomics on 706 baseline CSF samples quantified the same set of target proteins evaluated in the EHBS cohort. Baseline amyloid PET was used to ascertain presence or absence of underlying Alzheimer’s disease pathology in CN individuals with positive amyloid PET identified as AsymAD and hypometabolism on baseline FDG PET was used to identify AsymAD individuals who may be closer to symptomatic disease. Standardized uptake value ratios (SUVR) for florbetapir (AV45) and FDG PET were determined by ADNI investigators as described (https://adni.loni.usc.edu/data-samples/adni-data/neuroimaging/pet/). Cut-off SUVR values were determined based on Youden index in ROC analyses for AV45 (>1.226) and FDG (<1.191) using results from individuals classified as CN and Dementia at baseline ADNI visit. Individuals classified as EMCI (early MCI), LMCI (late MCI), or SMC (subjective memory complaint) were not included in the ROC plot to avoid inclusion of potentially ambiguous classifications. Three groups were identified in the ADNI cohort—CN/BM− (AV45 ≤ 1.226; *n* = 203), AsymAD (CN; AV45 > 1.226; *n* = 52) and Alzheimer’s disease (Dementia or MCI; AV45 > 1.226; *n* = 250). From these labelled groups, a subset of individuals matched for age, sex, race and education were identified. This finally resulted in 52 subjects for each of the three groups (CN/BM−, AsymAD and Alzheimer’s disease). Individuals without AV45 or FDG data and individuals with Dementia or MCI with AV45 SUVR ≤ 1.226 (*n* = 201) were not included in the analysis. Peptide panel identified in the EHBS cohort was tested to discriminate between CSF from CN/BM− controls and Alzheimer’s disease. The peptide panel was also assessed for ability to discriminate between AsymAD individuals with positive (SUVR <1.191; *n* = 10) or negative (SUVR ≥1.191; *n* = 42) baseline FDG PET scans in the ADNI cohort.

### Peptide selection to discriminate healthy controls and Alzheimer’s disease cases

**Figure 1:**
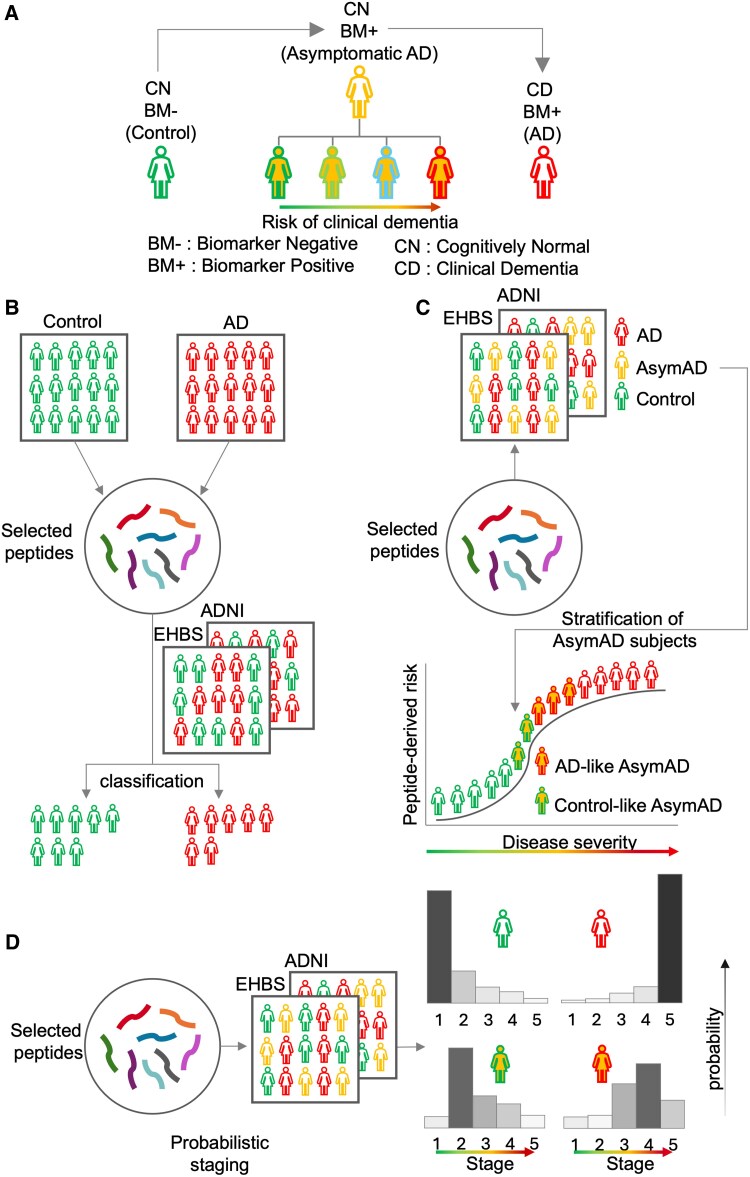
**Overview of data pipeline and analysis used to identify cerebrospinal fluid (CSF) peptides that best stratify asymptomatic Alzheimer’s Disease (AsymAD) conversion to symptomatic Alzheimer’s Disease.** (**A**) This work stratifies risk of developing clinical dementia in AsymAD subjects. AsymAD is seen as an intermediary stage between Control and Alzheimer’s disease, where the subjects are cognitively normal but biomarker positive (CN/BM+). Biomarker status is determined using the tTau:Aβ_42_ ratio in the EHBS data. The ASymAD class of CN/BM + subjects show variability in the development and onset of symptomatic clinical dementia defined as Alzheimer’s disease. The present work attempts to stratify this risk. (**B**) CSF peptide selection to discriminate between control and Alzheimer’s disease subjects. The selection is done using a supervised machine learning approach using a subset of the Emory Healthy Brain Study (EHBS) data. The identified peptide panel is then validated on a held-out subset of EHBS participants and in an external Alzheimer’s Disease Neuroimaging Initiative (ADNI) cohort. (**C**) The same peptide panel is useful in stratifying the AsymAD subjects for risk of cognitive decline and progression to symptomatic Alzheimer’s disease. The stratification of AsymAD subjects is based on their proximity to other subject classes –namely, Control or Alzheimer’s disease. This results in two sub-categories of AsymAD subjects—Control-like AsymAD and Alzheimer’s disease-like AsymAD. The peptide panel was found useful to stratify risk in AsymAD subjects from the ADNI cohort. (**D**) The progression risk from the peptide panel is also assessed using an independent approach that uses a probabilistic model for risk staging. The probabilistic model stages subjects for disease severity using cross-sectional data. Higher stages imply greater disease severity and risk. The model is trained using EHBS data from Control subjects that are cognitively normal biomarker negative (CN/BM−) and confirmed Alzheimer’s disease subjects that are cognitively symptomatic. The trained model is then used to infer stages for AsymAD in EHBS and all subjects in the external ADNI cohort. The inferred stages for the AsymAD sub-populations agree with their stratification. The overall results show that the peptide panel can stratify the disease risk in AsymAD subjects in two different datasets (EHBS and ADNI). This is also validated using two different methods (subfigure **C** and **D**).

Figure 1 illustrates the overall workflow of the study. CSF protein changes associated with Alzheimer’s disease from integrated discovery proteomics of brain and CSF were recently reported.^17^ In the current analyses, levels of 75 targeted peptides were examined and mapped to 58 unique proteins quantified by selected reaction monitoring mass spectrometry methods as detailed elsewhere.^21^ To identify peptides differentiating CN/BM− controls from patients with Alzheimer’s disease, a machine learning strategy of backward selection was employed using 80% of all CN/BM− and Alzheimer’s disease individuals from the EHBS cohort. A linear classifier, Support Vector Machine, first used all available peptides to distinguish Alzheimer’s disease cases from CN/BM− controls. Recursive feature elimination (RFE)^25^ eliminated the least informative peptides in a stepwise fashion to arrive at a smaller subset most important for the classification task. RFE-based biomarker selection outperforms other biomarker selection methods from proteomic datasets in supervised settings.^26^ The size of the subset at which to stop the recursive process is a user-defined parameter and was set to 14. The set of peptides resulting from RFE is not invariant to the choice of the classifier model. To address this, RFE was combined with two different classifiers (logistic regression and Support Vector Machine), which resulted in two different peptide subsets for classifying CN/BM− controls and Alzheimer’s disease cases. The final set of selected peptides was the intersection of the two subsets. Using the intersection provided a more stable and compact set of peptides for classification. Eighty per cent of data (CN/BM− and Alzheimer’s disease cases chosen randomly) were used to identify peptides, and 8 peptides were chosen and validated in the held-out set (remaining 20% data). The held-out data set played no role in peptide identification or classifier training. These peptides were also tested in a permutation test setting where the performance of the chosen peptides was compared to the performance of 100 000 randomly chosen peptide sets of the same size (*n* = 8). Correlation analyses between all measured peptides and MoCA score was performed using Kendall–Tau correlation to assess the strength of monotonic association between the peptide and MoCA. Finally, the RFE identified subset of proteins using the EHBS cohort was subsequently validated in separate dataset, the ADNI cohort. Labels for ADNI cases were determined using AV45 and FDG PET (as described in the subsection ‘Alzheimer’s disease neuroimaging initiative’ in Methods). Due to the smaller sample size of the ADNI cohort, a 6-fold cross-validation technique was utilized to evaluate the classification performance.

### Stratify AsymAD cases using proximity to control and Alzheimer’s disease peptide expressions

The 8 peptides chosen to discriminate between CN/BM− controls and Alzheimer’s disease were used to subcategorize AsymAD with more resolution. The initial stratification was performed using the EHBS cohort data and repeated with the ADNI cohort to validate the predictive accuracy and generalizability of the peptide subset. Specifically, a low-dimensional representation was used to stratify AsymAD cases. The representation involved two successive steps of dimensionality reduction. First, 8 peptides were selected from 75 peptides (as described in the subsection ‘Peptide selection to discriminate healthy controls and Alzheimer’s disease cases’ in Methods). Second, the t-distributed Stochastic Neighbor Embedding algorithm reduced the 8 peptides into 2 features. The analysis enabled a 2-dimensional visualization of how high-dimensional peptide data varies across subjects. Lastly, the AsymAD cases were categorized as ‘Control-like’ or ‘Alzheimer’s disease-like’, depending on which class (CN/BM− or Alzheimer’s disease) shares greater proximity with a given AsymAD case. This proximity is calculated using the k-Nearest Neighbor (k-NN) algorithm (k = 5). An AsymAD case is called ‘Control-like’ if the majority of its 5 nearest neighbors are CN/BM− and ‘Alzheimer’s disease-like’ otherwise. The *APOE* genotypes of the resulting AsymAD sub-categories were analysed for differences using the Fisher’s exact test.

### Model evaluation of disease progression risk with cross-sectional data

In the absence of available longitudinal data, cross-sectional disease progression data can be used to examine the consistency of the selected 8 peptide panel to predict risk of Alzheimer’s disease progression. The event-based model (EBM) is a probabilistic model which stages subjects for their varying disease severity using cross-sectional data.^27-30^ The underlying premise of EBM is that earlier changing biomarkers will show abnormal levels in a greater fraction of the population. Previously, EBM was successfully applied to model disease progression from cross-sectional observations in diverse neurodegenerative disorders, including Alzheimer’s disease,^27-31^ Parkinson’s Disease,^32^ Huntington Disease,^33^ and Multiple Sclerosis.^34^

Here, EBM was employed to examine the risk of ‘control-like’ and ‘Alzheimer’s disease-like’ AsymAD subjects transitioning to clinical dementia. EBM was applied to peptide expression data from CN/BM− and Alzheimer’s disease cases in the EHBS cohort. To ensure an unbiased validation, the EBM is not exposed to any data from EHBS AsymAD subjects or any participants in the ADNI study during training. The trained EBM infers the disease severity from peptide expression in AsymAD cases in the EHBS cohort, and for all subjects in the ADNI cohort. The EBM methodology assumes changes of biomarkers with disease progress are predominantly monotonic. Analysis of our 8 selected peptides showed that 5 (AQALEQAK, IASNTQSR, LGADMEDVCGR, VVSSIEQK, YDNSLK) demonstrate clear monotonic behaviour across diagnostic groups.

The scaled instantiation of the EBM was used in this analysis due to its computational benefits.^29^ The model was only trained on data from controls (CN/BM−) and Alzheimer’s disease subjects in the EHBS cohort. Only the peptides shown to discriminate controls and Alzheimer’s disease subjects in EHBS were used for model training. Hyperparameter selection was as follows: implicit feature exclusion parameter = 0; clustering hyperparameter = 4; cluster size = 2. The model used Markov Chain Monte Carlo with metropolis algorithm to generate samples of the biomarker abnormality event ordering. Of the 5 × 10^5^ iterations from Markov Chain Monte Carlo, the first 3 × 10^5^ were discarded as burn-in, and the last 2 × 10^5^ iterations were retained. The model was initialized using greedy search from 30 random starting points, each run for 800 iterations. The trained model inferred disease severity in AsymAD subjects in the EHBS cohort, and in all subjects in ADNI (Control/AsymAD/Alzheimer’s disease). In the ADNI cohort, each peptide’s distribution (modelled as a mixture of Gaussians by scaled instantiation of the EBM) was recalibrated to account for distribution shifts across cohorts. Subjects in both datasets were matched for age, gender and race. Statistical analysis was performed at an alpha of 0.05 to examine that the 8 predicted peptides could be used to successfully stratify patients using external cross-sectional cohort data.

### Statistical analysis

**Figure 2:**
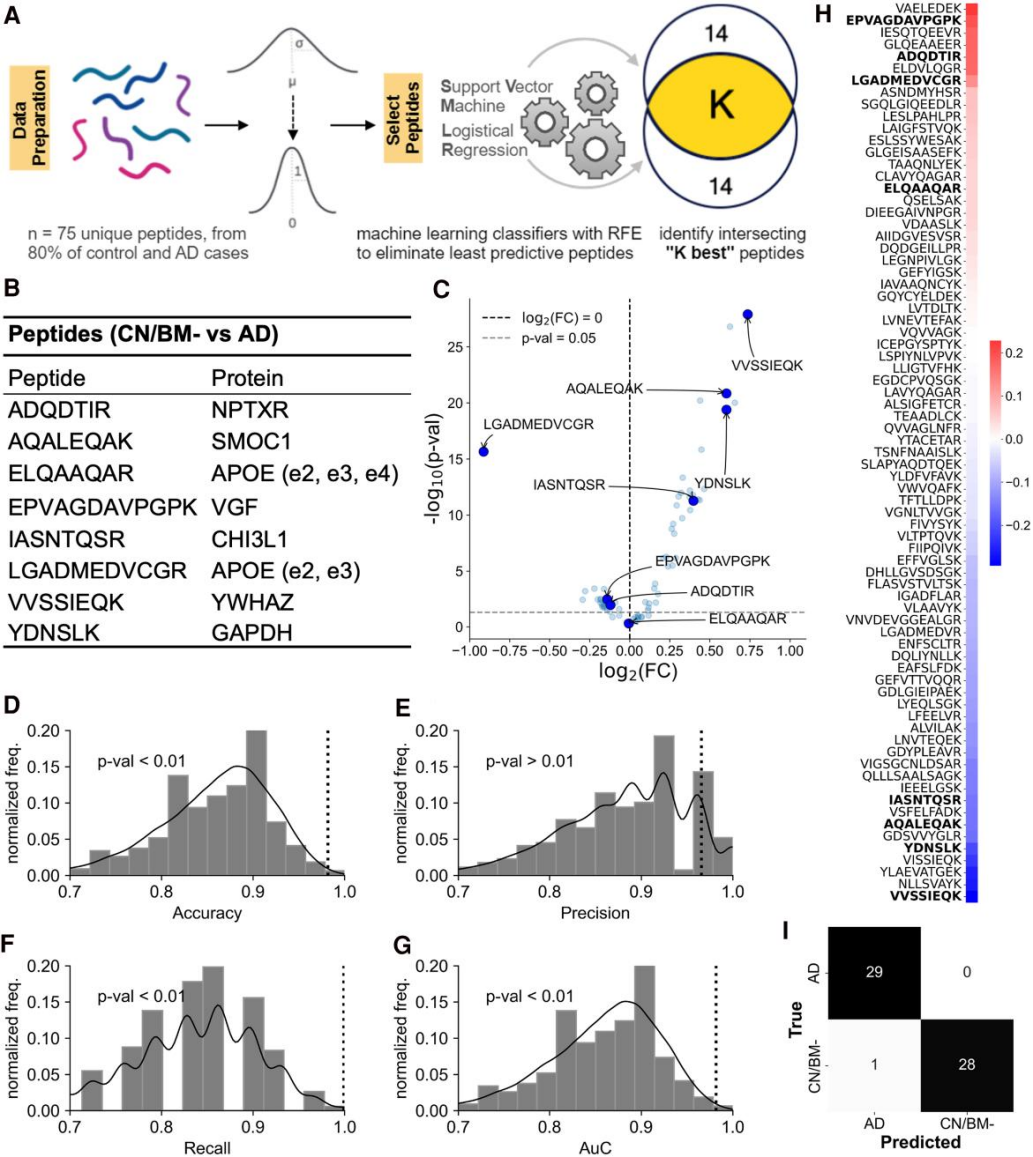
**Selection and evaluation of peptides for classifying biomarker-negative controls (CN/BM−) and symptomatic Alzheimer’s disease cases.** All analysis presented uses control and Alzheimer’s disease cases in the EHBS cohort. (**A**) Schematic describing the process of peptide selection. The peptide selection procedure uses only the training dataset (80% of the controls and Alzheimer’s disease cases) and starts by mean centreing and scaling the data to unit variance. Following this, recursive feature elimination (RFE) is run for peptide selection with two classifiers, support vector machine (SVM) and logistic regression, in an independent fashion, which results in two sets of selected peptides. In each case, the RFE stopping criterion is set to 14 peptides. The peptides intersecting between these two sets are chosen in the final set. (**B**) The 8 peptides (and their associated proteins), which are chosen via the RFE approach applied to the control and Alzheimer’s disease cases forming the training set, (**C**) A volcano plot showing log_2_FC (fold-change) versus −log_10_  *P-*value (Mann–Whitney *U* test) in the control and Alzheimer’s disease cases forming the held out set (*n* = 58). (**D–G**) Results from the random permutation test. 100 000 random sets of 8 peptides are generated and their performance on classifying the held out set of control and Alzheimer’s disease cases (*n* = 58) is evaluated using a logistic regression model. This is compared to the performance of the 8 peptides chosen via the RFE method (shown by the vertical dotted line). The metrices compared are accuracy, precision, recall, and receiver operating characteristic area under the curve (ROC-AUC). *P*-values are computed as the fraction of random peptide sets, which perform as good or better than the 8 peptides chosen via the RFE method. For all scores except precision, the scores from the RFE derived peptides show *P* < 0.01. (**H**) The Kendall’s–Tau correlation coefficient of the measured peptides with the Montreal Cognitive Assessment (MoCA) score (*n* = 392, including all control, AsymAD and Alzheimer’s disease cases). When the peptides are sorted for the correlation coefficient, the 8 peptides in **B** are found to lie near the extremes, indicating their stronger association with cognitive function. (**I**) Results from a logistic regression classifier on held-out control and Alzheimer’s disease cases (*n* = 58) that played no role in peptide selection or model training. The classifier trained on the 8 peptide panel only misclassified one CN/BM− as Alzheimer’s disease.

Statistical differences between the AsymAD and CN/BM− groups were evaluated using the McNemar-Bowker’s test for categorical variables and by paired *t*-test or Wilcoxon signed rank test for continuous variables depending on the distribution (Table 1). Correlation analyses between peptides and MoCA scores were performed using Kendall–Tau correlation to assess the strength of monotonic associations (Fig. 2H). For comparisons across disease groups (Supplementary Fig. 1), *P*-values were computed using Kruskal–Wallis test with *post hoc* comparisons made using FDR correction. Differences in APOE profiles between AsymAD subgroups were analysed using Fisher’s exact test. For the EBM analysis, statistical significance of differences in assigned disease stages was assessed using Chi-square test. All statistical tests were performed with significance level α = 0.05.

## Results

### Cohort characteristics

Table 1 shows characteristics of the full EHBS cohort (*N* = 1149) as well as three groups of individuals with symptomatic Alzheimer’s disease, AsymAD and CN/BM− controls (*N* = 134 each) matched for age, sex and race. The CN/BM− controls and AsymAD cases were also matched for education. As expected, the Alzheimer’s disease group was substantially different from both AsymAD and CN/BM− control groups in education, MoCA score, *APOE* ε4 allele frequency and levels of Aβ42, tTau and pTau (comparison across groups shown in Supplementary Fig. 1). *P*-values listed in Table 1 are for comparisons between AsymAD and CN/BM− control groups only. There are significantly higher *APOE* ε4 allele frequency, lower levels of Aβ42 and higher levels of tTau and pTau in AsymAD compared to CN/BM− controls (*P* < 0.0001 for all).

### Identification of CSF peptides associated with Alzheimer’s disease

Predictive CSF peptides were initially identified using the EHBS cohort data. Prior work identified changes in networks of brain-derived proteins in the CSF that discriminate between CN controls and patients with Alzheimer’s disease.^17,20^ Multidimensional scaling analysis of a small set of CSF samples revealed differences that segregated CSF samples into Alzheimer’s disease-like and Control-like groups.^17^ These results suggest that changes in specific proteins may allow stratification of AsymAD individuals into groups that are at higher or lower risk of transitioning to symptomatic Alzheimer’s disease. Using a targeted panel of 75 peptides that discriminate Alzheimer’s disease and Control CSF,^21^ machine learning-based feature selection algorithms identified a set of peptides that distinguished CN/BM− controls from symptomatic Alzheimer’s disease cases. Levels of these peptides in AsymAD CSF were evaluated by a series of unsupervised and supervised learning algorithms to determine their proximity to CN/BM− controls or Alzheimer’s disease cases. Finally, AsymAD individuals were stratified into those who more resemble CN/BM− controls or symptomatic Alzheimer’s disease.

Figure 2A shows a schematic for peptide biomarker selection using the machine learning strategy of RFE.^25^ The peptide biomarkers were identified by using RFE with two different linear classifiers (Support Vector Machine and logistic regression). Only those peptides which appeared in both selections (e.g. the union) were kept. The training set for peptide selection used 80% of the CN/BM− control and Alzheimer’s disease cases from the EHBS cohort. The selected peptides were validated on the held-out 20% of the EHBS cohort. The selected peptides are shown in Fig. 2B. A volcano plot shows log_2_FC (fold-change) versus −log_10_  *P-*value (Fig. 2C) in the control and Alzheimer’s disease cases forming the held-out set. These peptides also performed well on random permutation tests that analysed their classification receiver operating characteristic area under the curve (ROC-AUC) compared with randomly chosen sets of peptides (Fig. 2D–G).

Figure 2H shows Kendall-Tau correlation between all peptides measured across all subjects (CN/BM−, AsymAD, Alzheimer’s disease) and the MoCA score. The Kendall–Tau correlation shows the strength of monotonic association between the peptides and the MoCA score; the coefficients are sorted in a decreasing order. The peptides that differentiate CN/BM− and Alzheimer’s disease cases (bolded in Fig. 2H) tend to appear on the extremes of the sorted correlation coefficients. These peptides also perform well in discriminating the held out control and Alzheimer’s disease cases using a logistic regression model (Fig. 2I, sensitivity = 1.00, specificity = 0.965 and ROC-AUC = 0.98). These results suggest that the peptides chosen using the RFE approach classify CN/BM− and Alzheimer’s disease cases with very high accuracy and are also strongly associated with cognitive ability.

### Stratification of AsymAD cases

**Figure 3:**
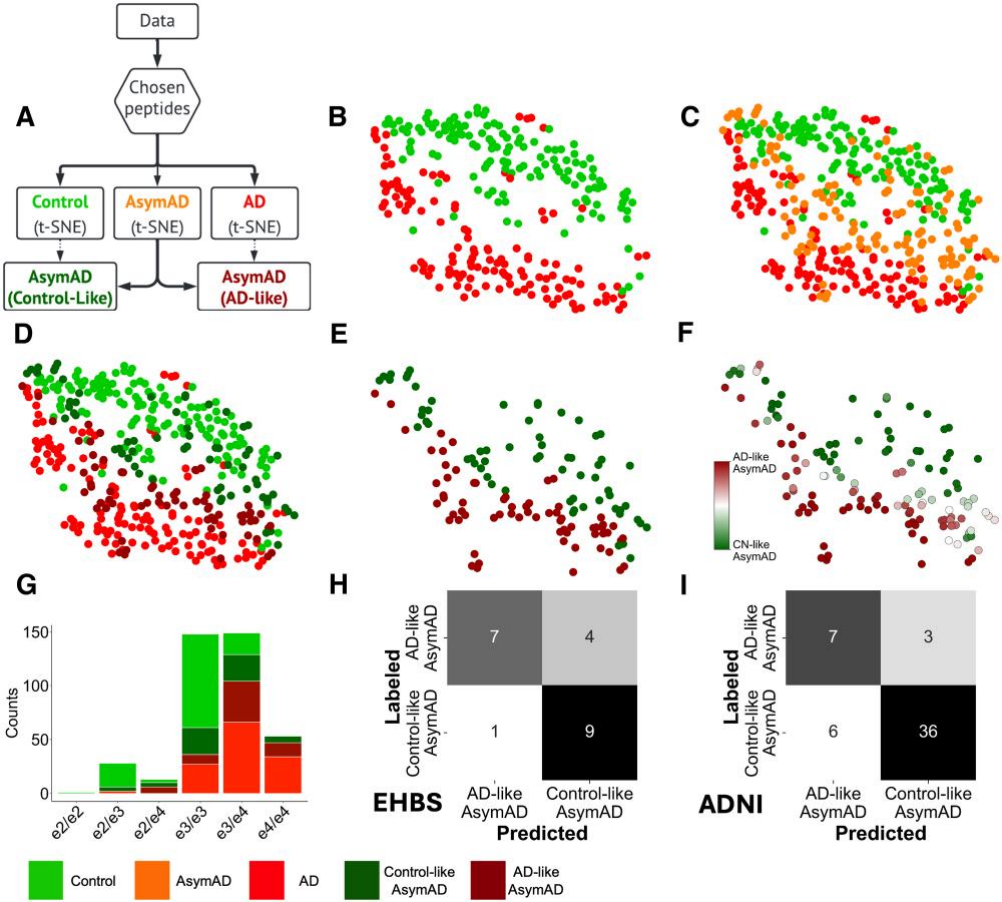
**Sub-categorization of asymptomatic Alzheimer’s disease (AsymAD) cases and evaluation of recursive feature elimination (RFE) selected peptides to classify subgroups.** (**A**) Schematic showing the overview to split asymptomatic Alzheimer’s disease subjects into ‘Control-like’ and ‘Alzheimer’s disease-like’ sub-categories. These sub-categories are not present in the original data and are derived using an unsupervised methodology by computing proximity of AsymAD subjects to well-defined biomarker-negative controls (CN/BM−) and Alzheimer’s Disease populations using the *k*-nearest neighbors (KNN) algorithm (*k* = 5). (**B**) Two-dimensional representations of CN/BM− control and Alzheimer’s disease subjects (*n* = 262) in the Emory Healthy Brain Study (EHBS) dataset using the t-distributed stochastic neighbor embedding (t-SNE) algorithm. These representations are computed using only the 8 peptides shown in Fig. 2B, which were predictive of the CN/BM− and Alzheimer’s disease groups. Hence, these low-dimensional representations are from two-levels of dimensionality reduction (peptide selection followed by t-SNE). A clear separation between the CN/BM− control and Alzheimer’s disease populations is noticeable. (**C**) AsymAD subjects overlaid with CN/BM− and Alzheimer’s disease subjects (*n* = 392, EHBS cohort). AsymAD subjects extend between the CN/BM− control and Alzheimer’s disease subjects and do not fall on a distinct, separable region. This observation is used to sub-categorize AsymAD cases. (**D**) AsymAD subjects with greater proximity (computed using the k-NN algorithm; *k* = 5) to Alzheimer’s disease or CN/BM− control subjects are defined as Alzheimer’s disease-like or Control-like, respectively (*n* = 392, EHBS). (**E**) The stratified AsymAD cases (Control-like and Alzheimer’s disease-like) shown for clearer visualization (*n* = 130, EHBS). (**F**) The AsymAD stratification shown in **B–E** depends on t-SNE initialization. To study this sensitivity to t-SNE initialization, the steps (**B–E**) in the analysis are repeated 100 times, and the KNN (*k* = 5) algorithm is used to stratify AsymAD individuals into Control-like or Alzheimer’s disease-like AsymAD as shown in **D** and **E**. The color bar shows the probability of a subject being assigned Control-like (dark green) or Alzheimer’s disease-like (maroon) subgroups (*n* = 130, EHBS). (**G**) Genotype profiles of all subjects (*n* = 392) in the EHBS cohort. The Alzheimer’s disease-like AsymAD cases (maroon) have a higher frequency of *APOE* ε4 allele, as compared to the Control-like AsymAD (dark green). This difference in ε4 allele frequencies is significant at the *P* < 0.001 threshold using Fisher’s exact test. (**H**) Confusion matrix showing classification performance of the peptide panel in Fig. 2B, on the held-out EHBS AsymAD subjects. A logistic classifier trained on AsymAD samples (*n* = 109) and tested on the held-out (*n* = 21) samples is shown here. (**I**) Confusion matrix showing classification performance of the peptide panel on the AsymAD samples (*n* = 52) in the Alzheimer’s Disease Neuroimaging Initiative (ADNI) cohort. A logistic regression is fitted in a six-fold cross-validation scheme and results from the held-out sets are pooled together (due to the smaller size of the AsymAD group in the ADNI dataset).

AsymAD cases were initially stratified using the EHBS cohort data. Figure 3 shows the low dimensional t-distributed Stochastic Neighbor Embedding analysis of the peptide data using the 8 RFE-selected peptides. Figure 3A shows the schematic of how AsymAD cases are sub-categorized into ‘Control-like’ and ‘Alzheimer’s disease-like’, based on their proximity to CN/BM− controls and Alzheimer’s disease cases, respectively.

Figure 3B–D shows the 2-dimensional representation of the peptide data derived using t-distributed Stochastic Neighbor Embedding algorithm in the EHBS cohort. The CN/BM− and Alzheimer’s disease cases occur in separable clusters (Fig. 3B, *n* = 262), and the AsymAD cases extend between them (Fig. 3C, *n* = 392). This spectral result is expected given that AsymAD individuals are hypothesized to be in a transitional stage between CN/BM− controls and symptomatic Alzheimer’s disease. Figure 3D and E shows stratification of AsymAD into Control-like and Alzheimer’s disease-like groups by using a k-NN (k = 5) algorithm in the EHBS cohort. Alzheimer’s disease-like AsymAD cases are those with ≥3 of 5 nearest neighbors among Alzheimer’s disease cases. Control-like AsymAD cases are those with the majority of nearest neighbors among CN/BM− controls. The low dimensional t-distributed Stochastic Neighbor Embedding representations were computed using only the 8 peptides selected by RFE for distinguishing CN/BM− from Alzheimer’s disease cases. The AsymAD sub-categories were compared for age, sex, race, education, cognitive performance and levels of CSF Aβ_42_, tTau and pTau (Supplemental Fig. 1). No significant difference was seen between the two AsymAD sub-categories for any of these features. In contrast, *APOE* profiles are significantly different (*P* = 0.0011 by Fisher’s exact test).

There is a significantly higher ε4 allele frequency in the Alzheimer’s disease-like AsymAD cases (Fig. 3G), which indicates a higher genetic risk for Alzheimer’s disease in these individuals. This result supports an associative link between AsymAD sub-categories and *APOE* genotype. It contrasts with the lack of difference between Alzheimer’s disease-like and Control-like subgroups in demographic features, cognitive performance or levels of CSF Aβ_42_, tTau or pTau (Supplemental Fig. 1). Together, the selected 8 peptides (ADQDTIR, AQALEQAK, ELQAAQAR, EPVAGDAVPGPK, IASNTQSR, LGADMEDVCGR, VVSSIEQK, YDNSLK) show a strong ability in classifying the AsymAD sub-categories (Fig. 3H) in the EHBS cohort (21 held out AsymAD samples). Figure 3I depicts the confusion matrix showing classification performance of the peptide panel for the AsymAD samples in the ADNI cohort (*n* = 52).

### Analysis of AsymAD subgroups using disease progression modelling

The RFE identified subset of 8 peptides was subsequently utilized to stratify cases from the ADNI cohort. Amyloid (AV45) PET results were used to determine the presence or absence of Alzheimer’s disease pathology. AV45 SUVR cut-off (>1.226) was determined based on ROC analysis of results from individuals classified as CN or Dementia at their baseline ADNI visit. ADNI subjects included for analysis were matched for age, sex, race and education (*n* = 52 subjects for each group of subjects).

**Figure 4:**
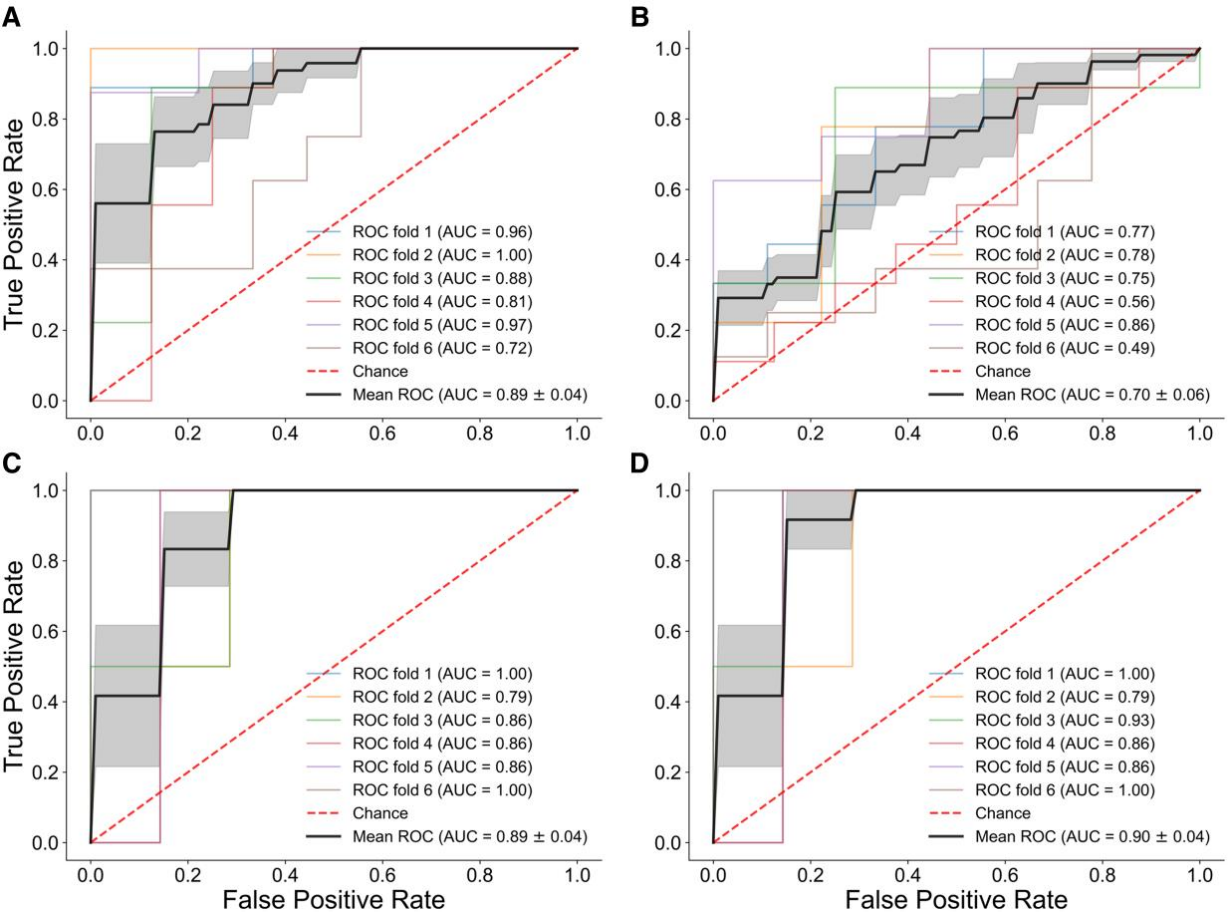
**Evaluation of selected peptide panel in Alzheimer’s Disease Neuroimaging Initiative (ADNI) data.** (**A**) Demographically matched biomarker-negative controls (CN/BM−, *n* = 52) and Alzheimer’s Disease (*n* = 52) individuals in the ADNI dataset were classified using peptides identified from the EHBS data. A mean receiver operating characteristic area under the curve (ROC-AUC) of 0.89 is observed. (**B**) Demographically matched CN/BM− (*n* = 52) and asymptomatic Alzheimer’s Disease (AsymAD, *n* = 52) individuals in the ADNI data, classified using the same peptide panel (mean ROC-AUC of 0.70). The classification performance remains stable when ApoE specific peptides are not used for classification (not shown). (**C**) AsymAD with positive and negative Fluorodeoxyglucose Positron Emission Tomography (FDG PET) was classified using the same peptide panel (mean ROC-AUC of 0.89). (**D**) Of the 8 peptides, the two ApoE specific peptides were not used. Using the remaining 6 peptides, the classification performance did not see a drop and remained stable with a ROC-AUC of 0.90. In all cases, a 6-fold cross-validation approach is used with a linear logistic regression model. The shaded region shows the standard error for the mean receiver operating characteristic (ROC).

Figure 4 shows the performance of the 8 peptides with the ADNI cohort. In the ADNI dataset, these peptides were used to classify CN/BM− controls (AV45 SUVR ≤1.226) and individuals with symptomatic Alzheimer’s disease (MCI or Dementia with AV45 SUVR >1.226). A 6-fold cross-validation approach using a linear logistic regression model showed a mean ROC-AUC of 0.89 (Fig. 4A). Next, CN subjects were classified, including the two categories of matched CN/BM− controls and AsymAD cases (CN with AV45 SUVR > 1.226). The 8 peptides classified these two groups with a mean AUC of 0.70 (Fig. 4B). Lastly, baseline FDG PET results were exploited to identify individuals with hypometabolism as a means of stratifying ADNI AsymAD individuals who might be closer to developing clinical symptoms. As was done with AV45 results, FDG PET SUVR cut-off (<1.191) was determined based on ROC analysis of results from individuals classified as CN or Dementia at their baseline ADNI visit. This cut-off identified 10 AsymAD cases with evidence of hypometabolism and 42 with normal FDG PET scans. Figure 4C shows the performance of the subset of peptides (*n* = 8) previously identified in the EHBS cohort, to discriminate between Control-like (FDG PET SUVR > 1.191) versus Alzheimer’s disease-like AsymAD (FDG PET SUVR < 1.191) cases in the ADNI cohort. Despite small sample sizes, the mean ROC-AUC for the ADNI dataset was 0.89. As in our sub-categorization of AsymAD cases in the EHBS cohort, demographic features (except gender), CSF Alzheimer’s disease biomarkers and MoCA score were not different in the FDG PET-positive and -negative AsymAD subgroups (Supplemental Fig. 2). These results support the predictive ability of RFE selected peptide panels to discriminate CN/BM− controls from Alzheimer’s disease cases and to sub-categorize AsymAD cases, respectively, in an independent dataset. Further, the predictive ability of the peptide panel was examined without its ApoE associated peptides. Of the 8 peptides, 2 were related to ApoE (LGADMEDVCGR and ELQAAQAR). Excluding these two from the panel, the smaller set of 6 peptides showed stable performance in classifying the AsymAD sub-categories with a mean ROC-AUC = 0.90 (Fig. 4D). This shows that the peptide panel does not hinge upon ApoE specific peptides to sub-categorize AsymAD cases.

### Validation of varying disease risk in AsymAD cases using disease progression modelling

**Figure 5:**
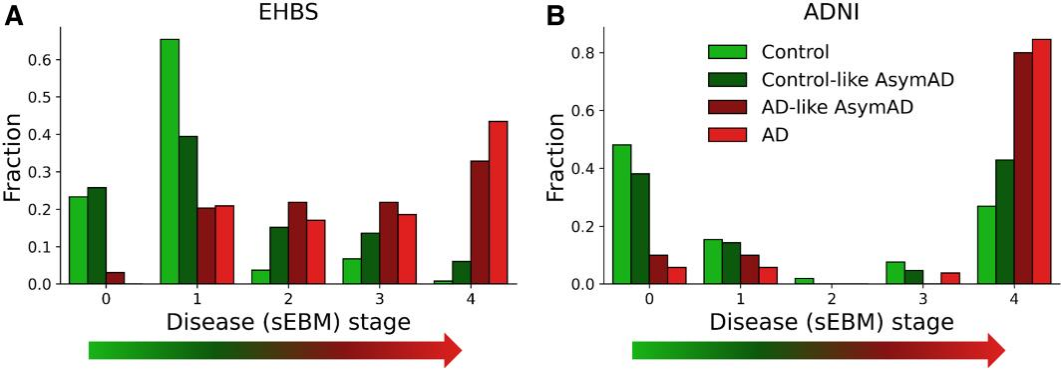
**Results from disease progression modelling across two different patient cohorts, Emory Healthy Brain Study (EHBS) and Alzheimer’s Disease Neuroimaging Initiative (ADNI).** Event-based modelling (EBM) is applied to stratify subjects in the Emory Healthy Brain Study (EHBS) shown on the left and the Alzheimer’s Disease Neuroimaging Initiative (ADNI) shown on the right. *x*-axis shows disease stage, which represents increasing disease severity. *y*-axis shows fraction of subjects in each disease group that were assigned the stage. The scaled Event Based Model (sEBM)^29^ is trained on EHBS peptide data to learn a disease progression trajectory, which is used to stage disease severity in subjects. Only cognitively normal biomarker negative (CN/BM−) control cases (*n* = 133) and cognitively symptomatic confirmed Alzheimer’s disease cases (*n* = 129) from EHBS are used to train the model using the peptide panel in Fig. 2B. The model inferred disease stages for asymptomatic Alzheimer’s Disease (AsymAD) in the EHBS cohort, and all subjects in the ADNI cohort. Thus, the model was never shown any AsymAD subject during training and was assessed on an external dataset (ADNI). (**A**) Results on the EHBS cohort. Assigned stages differentiate for disease labels (*n* = 392, *P*-value < 1 × 10^−15^). The control-like AsymAD (*n* = 64) are in lower stages compared to the Alzheimer’s disease-like AsymAD (*n* = 66) cases (*P* < 1 × 10^−5^). (**B**) Results on the ADNI cohort. Assigned stages differentiate for disease labels (*n* = 156, *P* < 1 × 10^−4^). The control-like AsymAD (*n* = 42) are assigned lower stages compared to the Alzheimer’s disease-like AsymAD (*n* = 10) cases (*P* < 0.05). No data from the ADNI cohort was used during model training. All *P*-values were derived using a chi-square test.

The stratification of Alzheimer’s disease-like and control-like AsymAD was examined in 2 cohorts (EHBS and ADNI) using discriminative classification methods (Figs 3 and 4). However, due to a lack of sufficient longitudinal data, progression trajectories of these subgroups cannot be studied in greater detail. To overcome this limitation, a type of disease progression modelling that leverages cross-sectional data was employed. The event-based model, or EBM,^27-30^ is a probabilistic model, which used cross-sectional data to construct a trajectory of disease progression and stage subjects for their disease risk (Fig. 5). Specifically, EBM^27-30^ was used to examine the risk of AsymAD subjects developing clinical Alzheimer’s disease. EBM has been previously used to successfully deduce progression patterns in diverse neurodegenerative conditions, including Alzheimer’s disease, Parkinson’s Disease and multiple sclerosis.^27-34^

The RFE identified panel of 8 peptides (Fig. 2B) was used to define an Alzheimer’s Disease staging system that has 5 stages. Higher stages represent patient states that are further along the disease progression trajectory. Subjects are assigned to these stages by the model in a probabilistic manner. Subjects with clinical dementia or those who have high risk to develop it are assigned higher stages. In our experiments, only CN/BM− and Alzheimer’s disease subjects from EHBS are used to build the progression model. All AsymAD cases in the EHBS cohort and all subjects in the ADNI cohort are only used during inference; thus, the model does not use the data from these subjects during training. If the 8 peptides are truly predictive of disease progression risk, it would be expected that the AsymAD sub-categories (i.e. Control-like and Alzheimer’s disease-like) would show a difference in their assigned EBM stages. Figure 5A shows that the AsymAD subgroups (i.e. Control-like and Alzheimer’s disease-like) are indeed assigned different disease stages in the EHBS cohort (*P* < 1 × 10^−5^). A similar pattern is seen in the ADNI cohort (Fig. 5B) where the Control-like and Alzheimer’s disease-like AsymAD cases show differences in their stages (*P* < 0.05) using a Chi-square test. The CN/BM− and Alzheimer’s disease cases in ADNI also show a strong separation (*P* < 1 × 10^−3^), even though their data were not used during model training. Supplementary Fig. 3 shows the positional variance diagram for the 8 peptides. Supplementary Fig. 4 shows the distribution of these peptides across the EBM inferred stages in the EHBS cohort, which tends to agree with the relative positions of these peptides in the positional variance diagram. Distribution of disease pathology markers (Aβ_42_, tTau, pTau and their ratios) across EBM inferred disease stages in the EHBS cohort are also shown in Supplementary Fig. 5. These markers were not used by the event-based model in disease staging. The analysis shows Aβ_42_ to be the earliest changing (shows significant differences between stages 0 and 1, *P* < 10^−4^). tTau and pTau levels show significant changes between stages 1 and 2. The model can correctly infer the early roles of these hallmark pathologies without being directly exposed to them.

Further, it is noteworthy that the labels in the ADNI cohort were assigned using AV45 and FDG-PET SUVR, which is different from how the labels were assigned in the EHBS cohort. In summary, the EBM results illustrate that the Alzheimer’s disease-like AsymAD subjects have a higher risk of progressing to clinical Alzheimer’s disease compared to control-like AsymAD. As such, there is evidence to indicate the 8-peptide panel can predict longitudinal disease risk of converting from a control or asymptomatic stage to clinical dementia. The predictive efficacy of the 8 peptides was independently observed in both datasets—EHBS and ADNI.

## Discussion

To our knowledge, this study includes one of the largest sets of CSF data from cognitive normal individuals and one of the largest cohorts of asymptomatic Alzheimer’s disease cases. The large sample size enabled machine learning approaches to identify novel putative biomarkers. Our findings demonstrate that CN individuals with CSF biomarkers indicating silent Alzheimer’s disease pathology (AsymAD) have distinct patterns of CSF peptide levels compared with Alzheimer’s disease biomarker-negative controls. Machine learning approaches successfully stratified AsymAD cases to identify sub-categories whose CSF peptide profiles are more ‘Alzheimer’s disease-like’ or more ‘Control-like’. Results showed consistent patterns across two independent cohorts and two independent machine learning approaches, including dynamic disease progression modelling with cross-sectional data. These results identify key features that may stratify individuals at differing risk of progression to symptomatic Alzheimer’s disease. Future longitudinal studies may allow prioritization of individuals for secondary prevention trials or treatment with emerging disease-modifying therapies.

The present study showed that a small set of 8 differentially expressed peptides can effectively distinguish Alzheimer’s disease cases from cognitively healthy controls: ADQDTIR, AQALEQAK, ELQAAQAR, EPVAGDAVPGPK, IASNTQSR, LGADMEDVCGR, VVSSIEQK and YDNSLK. Importantly, the set of predictive peptides have been shown in recent studies to be valuable in tracking disease status and progression. Neuronal pentraxin receptor (NPTXR) isoform 1 (protein for the ADQDTIR peptide) has been shown to be a CSF biomarker of Alzheimer’s disease progression^35^ with levels differing between MCI and more advanced Alzheimer’s disease stages. YWHAZ (protein for the VVSSIEQK peptide) has recently emerged as an important biomarker to discriminate Alzheimer’s disease from non-Alzheimer’s disease cases with cognitive impairment and also predicts individuals with high Tau and low Aβ42 levels.^36^ CHI3L1 (protein for the IASNTQSR peptide; also known as YKL-40) has been reported in other studies as a potential prognostic fluid biomarker, and its ratio to Aβ42 is predictive for developing cognitive impairment.^37^ CHI3L1 is also a glial/inflammation related biomarker.^17,36,38^ VGF (protein for the EPVAGDAVPGPK peptide) has been strongly associated with cognitive trajectory and suggested to act through mechanisms independent of amyloid plaques and neurofibrillary tangles in contributing to cognitive decline.^39^ Further, VGF has also been identified as a key regulator playing a causal role in protecting against Alzheimer’s disease pathogenesis and progression.^40^ SMOC1 (protein for AQALEQAK peptide), which is related to the extracellular matrix and strongly correlated with global Alzheimer’s disease pathology in brain,^41^ has shown the ability to discriminate between Alzheimer’s disease and non-Alzheimer’s disease cognitive impairment (specificity for Alzheimer’s disease) and to predict levels of CSF Aβ42, tTau and pTau.^36^ GAPDH (protein for the YDNSLK peptide) is known to form stable aggregates with extracellular Aβ, and these aggregates have been found to be proportional to the progressive stage of Alzheimer’s disease.^42,43^

These 8 peptides, each with plausible biological connection to Alzheimer’s disease pathophysiology, were found to be among the most strongly associated with cognition and were able to discriminate CSF samples from patients with Alzheimer’s disease and Controls with 98% accuracy (Fig. 2H and I). This ability of the peptide panel to classify patients with Alzheimer’s disease and Controls is also seen in the ADNI cohort, with a mean ROC-AUC of 0.89 for subjects, which were demographically matched for age, sex, race and education (Fig. 4A).

The 15–20 year period during which Alzheimer’s disease neuropathology evolves silently prior to cognitive decline offers a window of opportunity to slow or prevent clinical disease. However, as many individuals with Alzheimer’s disease neuropathology never develop symptoms during life,^12,13^ it is critical to develop tools that identify individuals at greatest risk of cognitive decline. The Alzheimer’s disease-like AsymAD cases show a higher frequency of the *APOE* ε4 allele but are otherwise indistinguishable from the Control-like AsymAD cases based on demographics, cognitive performance, or level of CSF Aβ_42_, tTau, or pTau (Supplemental Fig. 1). The ApoE-associated peptides selected by RFE can discriminate presence or absence of the ε4 isoform. *APOE* genotype is firmly established as the strongest genetic risk factor for Alzheimer’s disease.^44,45^ The selection of ApoE-related peptides and higher frequency of the ε4 allele in the Alzheimer’s disease-like subgroup (Fig. 3G) supports the possibility that these individuals may be at greater risk of progression.

To further test the peptides identified in the EHBS cohort, targeted proteomics analysis was performed on 706 baseline ADNI CSF samples. Since amyloid PET scans were available for the ADNI cohort, AV45 PET positivity was used as a means of defining individuals with underlying Alzheimer’s disease pathology. The 8 RFE-selected peptides were effective in discriminating between CN/BM− (AV45 PET negative) controls and amyloid PET-confirmed Alzheimer’s disease with mean AUC of 0.89 (Fig. 4A). Among all CN individuals in ADNI, there were 52 with asymptomatic Alzheimer’s disease based on positive amyloid PET. The 8 RFE-selected peptides were able to discriminate these AsymAD cases from a demographically-match cohort of 52 CN individuals with negative AV45 PET scans with a mean area under the curve (AUC) of 0.70 (Fig. 4B). Only a very small number of individuals in ADNI have transitioned from CN to MCI or Dementia during longitudinal follow up. In addition to being a rare event, clinical progression is complicated by frequent reversions from MCI to CN.^46-48^ To avoid these limitations, FDG PET was used to identify AsymAD individuals with evidence of hypometabolism who may be at greater risk of symptomatic progression. Despite small sample size (*n* = 10), the 8 RFE-selected peptides classified FDG-positive AsymAD cases with a mean AUC of 0.89 (Fig. 4C). This predictive ability does not hinge upon ApoE-associated peptides, as is seen from the ability of non-ApoE peptides to sub-categorize the AsymAD subjects in the ADNI cohort (Fig. 4D).

Further analysis using event-based modelling for disease progression confirms that the AsymAD subgroups have varying risks of developing clinical dementia (Fig. 5). The disease progression model was constructed only using CN/BM− and Alzheimer’s disease cases in EHBS. Yet, it staged the AsymAD participants in EHBS and ADNI in accordance with their stratified levels (Control-like and Alzheimer’s disease-like). Disease progression modelling with cross-sectional data provided an independent methodological validation of the constructed 8 peptide panel to effectively stage AsymAD cases. In the absence of currently available longitudinal data, the innovative use of cross-sectional data with a state-of-the-art dynamic disease progression model (e.g. EBM) lends further credence to the ability of the identified 8 peptides to proactively predict patient risk.

Unlike our previous studies with deep proteomics and network analyses,^17,20^ the purpose of the current work was to evaluate CSF peptides that might serve as biomarkers to predict cognitive decline in CN individuals harbouring Alzheimer’s disease pathology. Deep proteomics comparing Alzheimer’s disease-like and Control-like AsymAD cases should produce better understanding of changes occurring during the transition from asymptomatic to symptomatic stages of Alzheimer’s disease.

There are some limitations to the underlying methodology and data. Specifically, the cross-sectional secondary validation method utilized the scaled instantiation of the EBM model,^29^ which like all current EBM methodologies, assumes biomarkers exhibit a monotonic trajectory. The majority, but not all, of the 8 predictive peptides illustrate clear monotonic behaviour. However, the key limitation to the present work is the lack of longitudinal data. Longitudinal follow-up of individuals with asymptomatic Alzheimer’s disease will be required for ultimate validation of predictive biomarkers. Fortunately, the design of the EHBS will be able to directly test putative predictive biomarkers over the course of time.

Future work includes the development of improved cross-sectional data methods that do not rely on a monotonic assumption, and of course, the collection and evaluation of longitudinal clinical data. Once longitudinally validated, the identified 8 peptides can prioritize individuals for prevention trials and treatment with emerging disease-modifying therapies for Alzheimer’s disease.

## Supplementary Material

fcaf121_Supplementary_Data

## Data Availability

Data for the Alzheimer’s Disease Neuroimaging Initiative (ADNI) is available https://adni.loni.usc.edu/data-samples/adni-data/ and for the Emory Healthy Brain Study (EHBS) is available by contacting James Lah at jlah@emory.edu. Finally, all code for this study can be downloaded at the Laboratory for Pathology Dynamics GitHub: https://github.com/pathology-dynamics/cogdec.git.
