# Supplementary material for: Stratifying risk of Alzheimer’s disease in healthy middle-aged individuals with machine learning
Source: Brain Commun. 2025 Mar 25;7(2):fcaf121. doi: 10.1093/braincomms/fcaf121 (PMC11986205; doi:10.1093/braincomms/fcaf121)
Supplement: fcaf121_Supplementary_Data [file fcaf121_supplementary_data.docx]

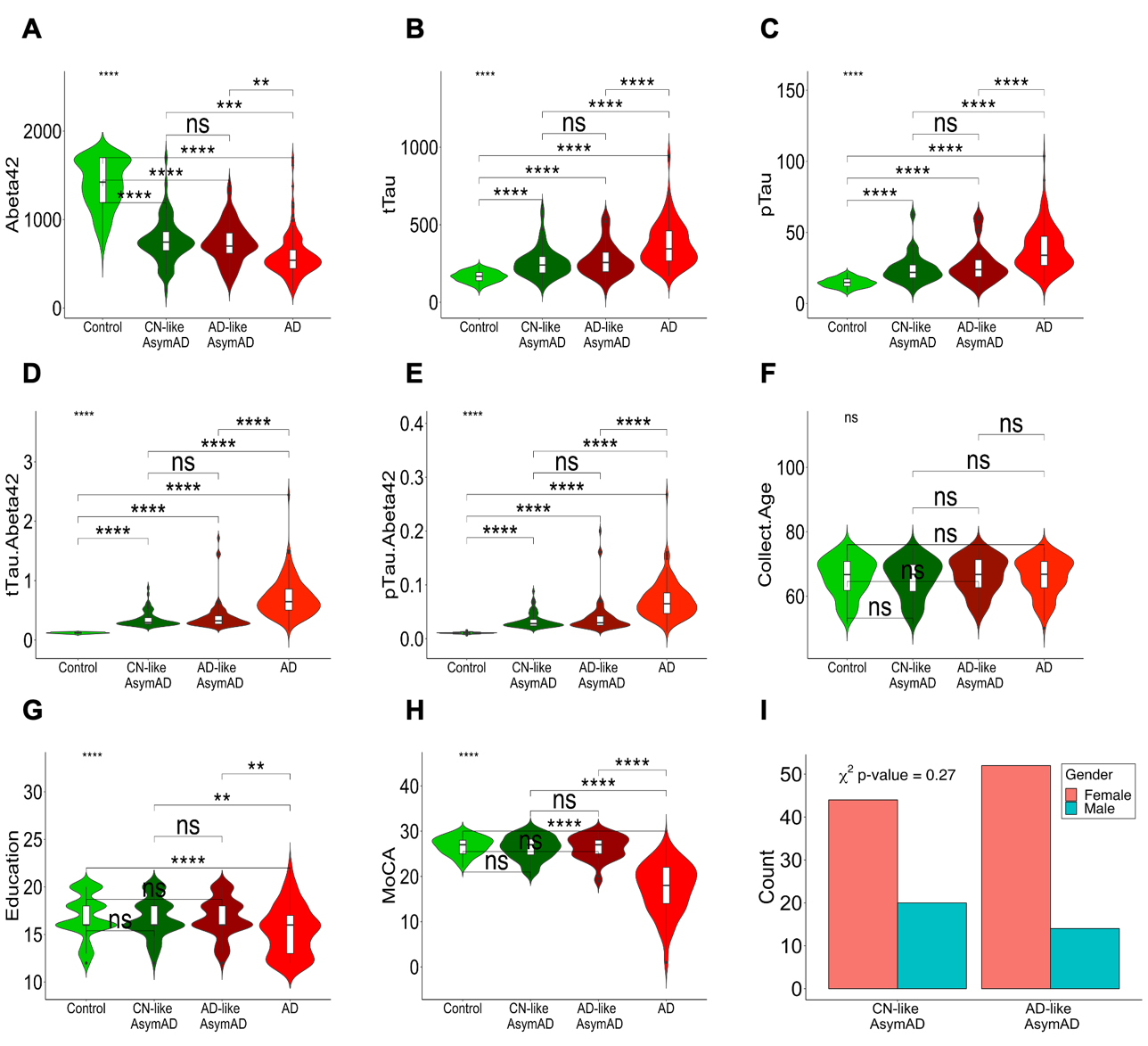


**Supplementary Figure S1** – Comparing Control-like AsymAD (n=64) and AD-like AsymAD (n=66) for CSF analytes (Aβ42, tTau, pTau, and their ratios) and demographics (age, sex, education, MoCA score). Additionally, these groups were also compared to controls (n=133) and AD cases (n=129). In all comparisons, no difference is seen between the Control-like and AD-like AsymAD subjects. (**a-e**) Comparison for Aβ_42_ (pg/mL), tTau (pg/mL), pTau (pg/mL), and ratios tTau:Aβ42 and pTau:Aβ42. (**f-i**) Comparing the demographics for age (years), education (years), MoCA score and gender. In subfigures **a-h**, p-values are computed using Kruskal-Wallis test, and post-hoc comparisons are made using FDR correction. The p value significance is indicated using stars and can be interpreted as follows: ns (p>0.01), *(p<0.01), **(p<0.001), ***(p<0.0001), ****(p<0.00001). Stars in the top left show p value results from the overall Kruskall-Wallis test. For subfigure **i**, comparison is done using the Chi-squared test. Overall, the results show no significant difference between the Control-like and AD- like AsymAD individuals when comparing for CSF analytes, demographics, or MoCA scores.


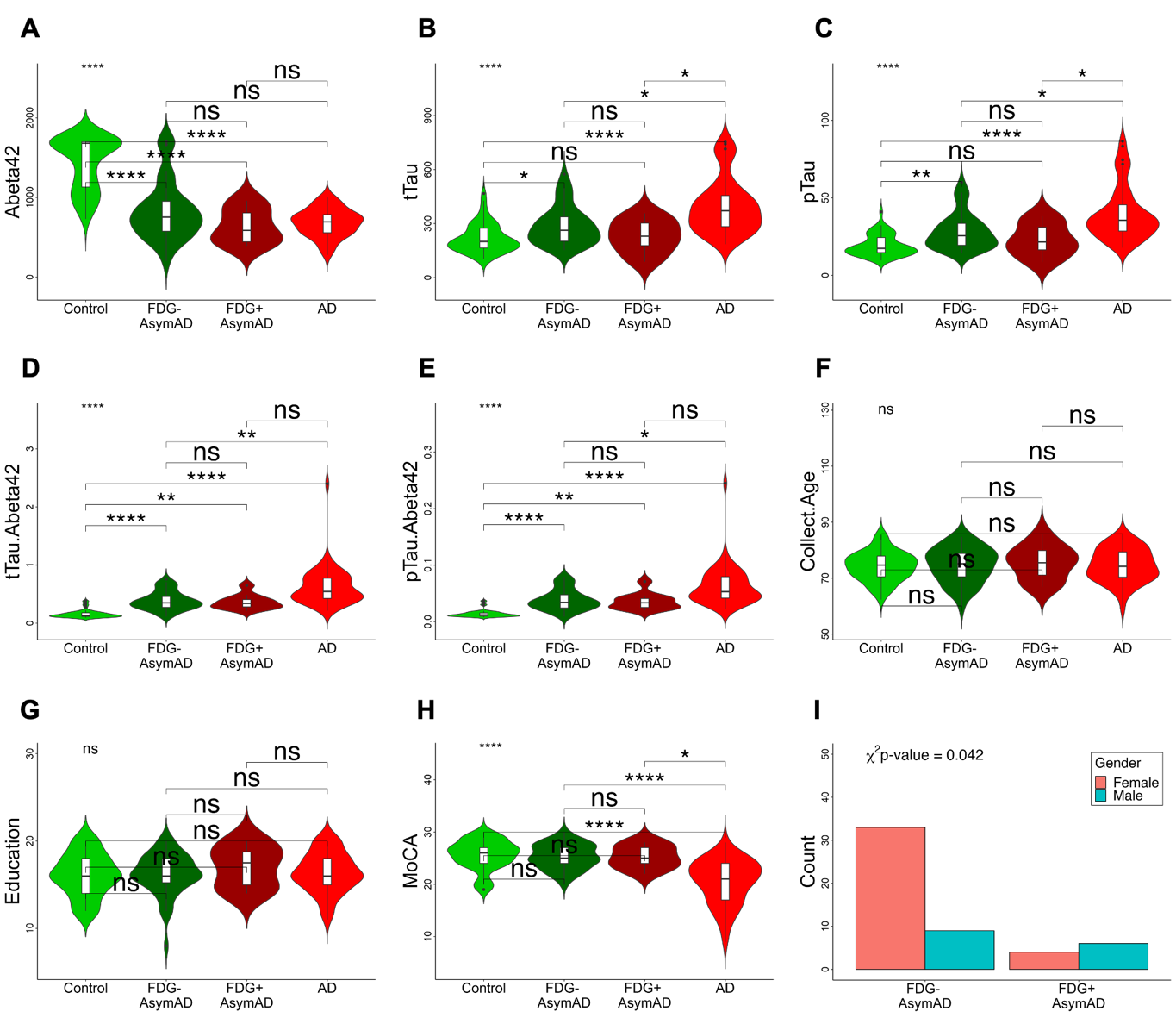


**Supplementary Figure S2** – Comparing FDG-negative AsymAD (n=42) and FDG-positive AsymAD (n=10) for CSF analytes (Aβ42, tTau, pTau, and their ratios) and demographics (age, sex, education, MoCA score). Additionally, these groups were also compared to controls (n=52) and AD cases (n=52). In all comparisons exept gender, no difference is seen between the two AsymAD subgroups. (**a-e**) Comparison for Aβ_42_ (pg/mL), tTau (pg/mL), pTau (pg/mL), and ratios tTau:Aβ42 and pTau:Aβ42. (**f-i**) Comparing the demographics for age (years), education (years), MoCA score and gender. In subfigures **a-h**, p values are computed using Kruskal-Wallis test, and post-hoc comparisons are made using FDR correction. The p value significance is indicated using stars and can be interpreted as follows: ns (p>0.01), *(p<0.01), **(p<0.001), ***(p<0.0001), ****(p<0.00001). Stars in the top left show p value results from the overall Kruskall-Wallis test. For subfigure **i**, comparison is done using the Chi-squared test. Overall, the results show no significant difference between the FDG- negative and FDG-positive AsymAD individuals when comparing for CSF analytes, demographics (except gender), or MoCA scores.

**
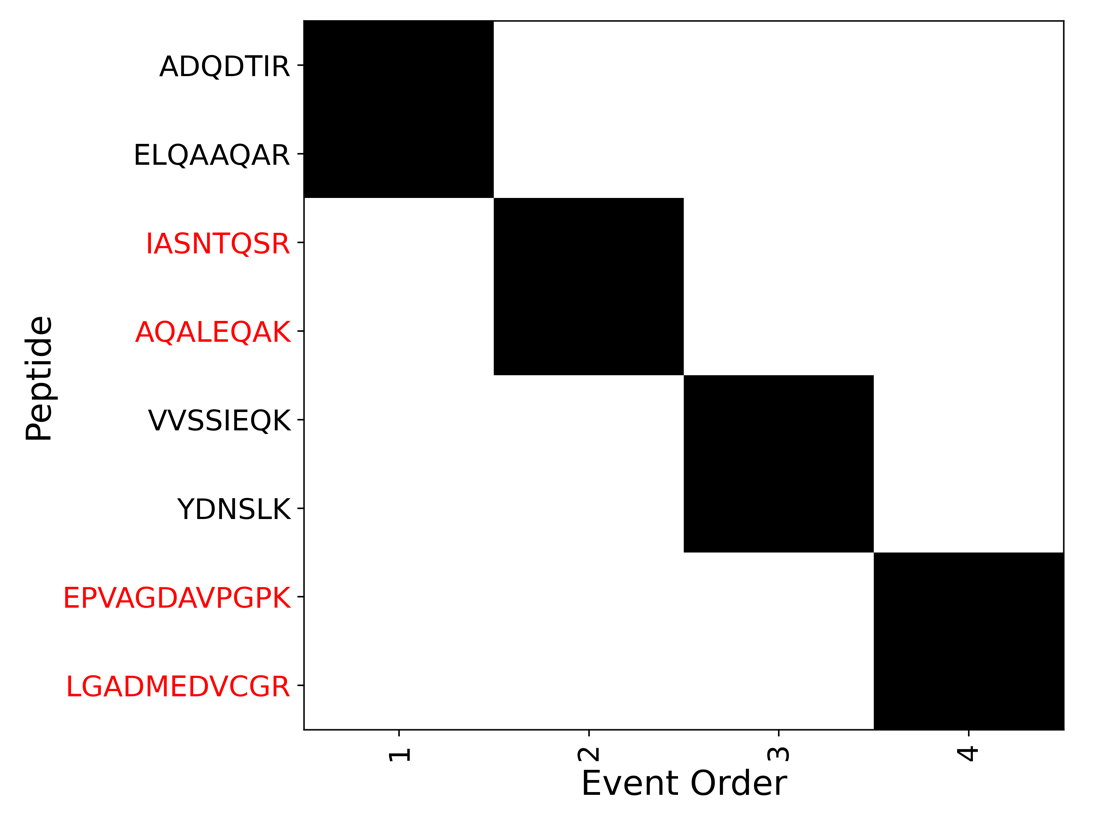
**


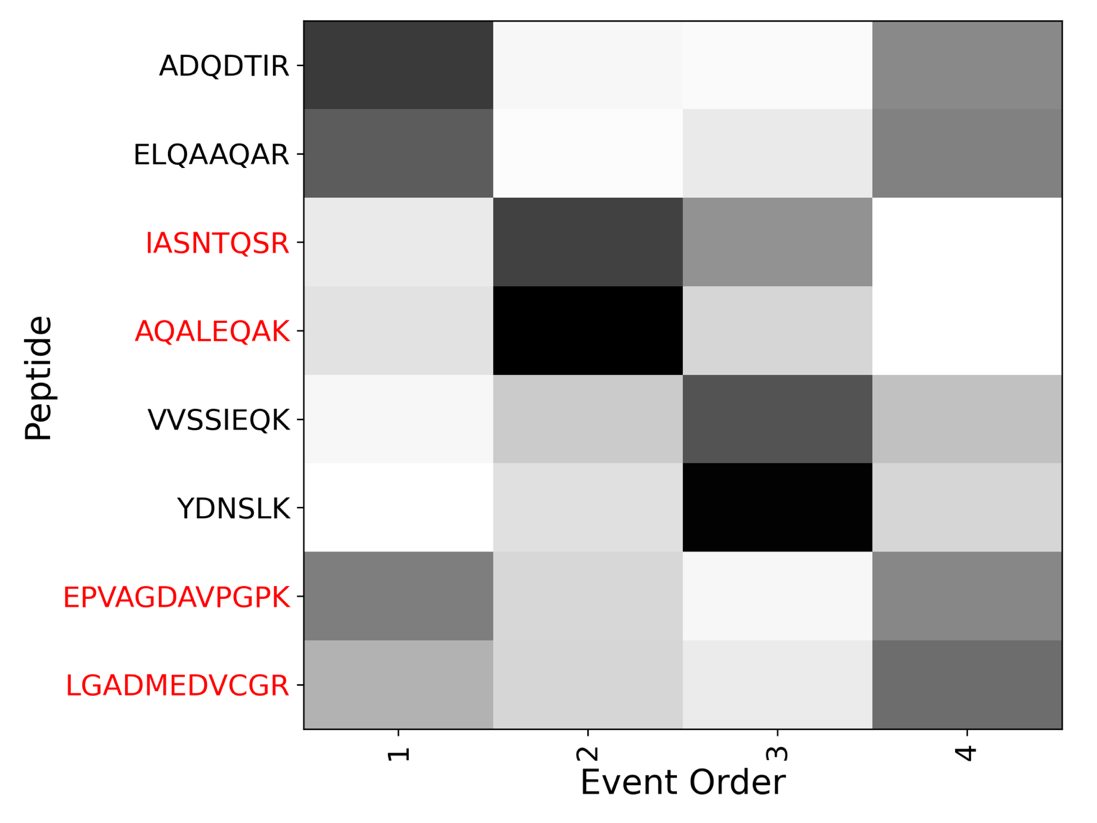


**Supplementary Figure S3** – (Top) Positional variance diagram for the peptides used in the event-based model, (bottom) Bootsrap positional variance diagram (number of bootstraps = 50). The model fitting process is detailed in section 2.4 (3x10^5^ MCMC samples, n = 262). The peptides ADQDTIR and ELQAAQAR are placed in the first cluster and are the earliest to show changes according to EBM. The next cluster of changing peptides is formed by IASNTQSR and AQALEQAK. The third cluster of changing peptides is formed by VVSIEQK and YDNSLK. Finally, the last cluster to change is formed by EPVAGDAVPGPK and LGADMEDVCGR. The positional variance diagram shows the variability in the positions occupied by these peptides. Darker shades represent a more certain position for any given peptide.

**
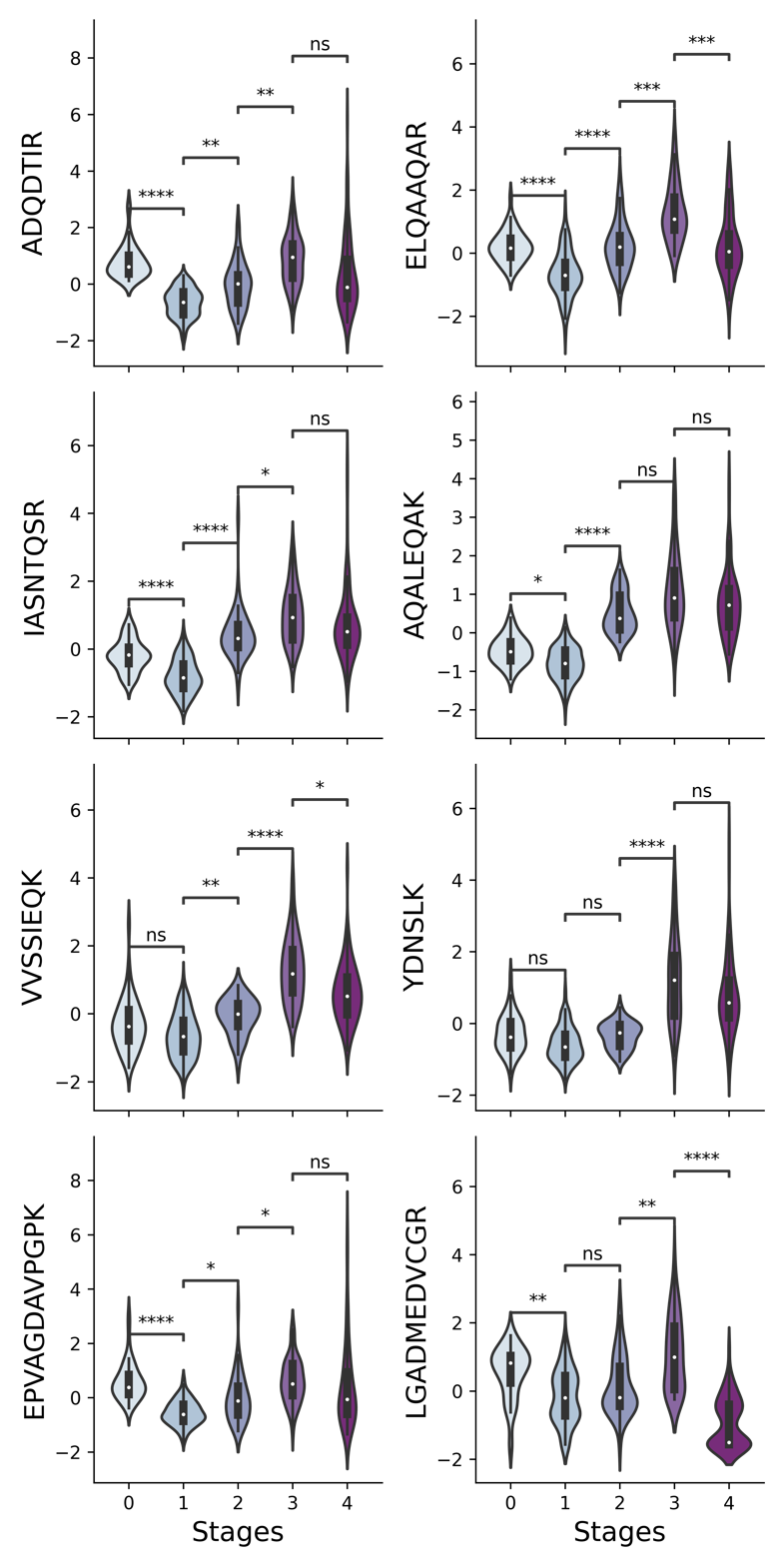
**

**Supplementary Figure S4**- The distribution for the 8 peptides as a function of disease stage inferred by EBM, across all subjects in the EHBS cohort (n=392). The top row peptides (ADQDTIR and ELQAAQAR) are assigned the first stage by the EBM model and show early changes (between stage 0 and 1). The second row consists of the peptides which were assigned the second stage cluster by EBM. These show significant differences between stage 1 and 2. The third stage peptides (third row) are the next to change. VVSSIEQK and YDNSLK show strongest changes between stages 2 and 3. Finally, EPVAGDAVPGPK and LGADMEDVCGR are the last 2 peptides to change according to the EBM model. P-values computed using Mann-Whitney test and corrected using the Holm-Bonferroni method (* p < 10^-4^, ** p < 10^-6^, *** p < 10^-8^, **** p < 10^-10^). The y-axis shows the normalized peptide levels.

**
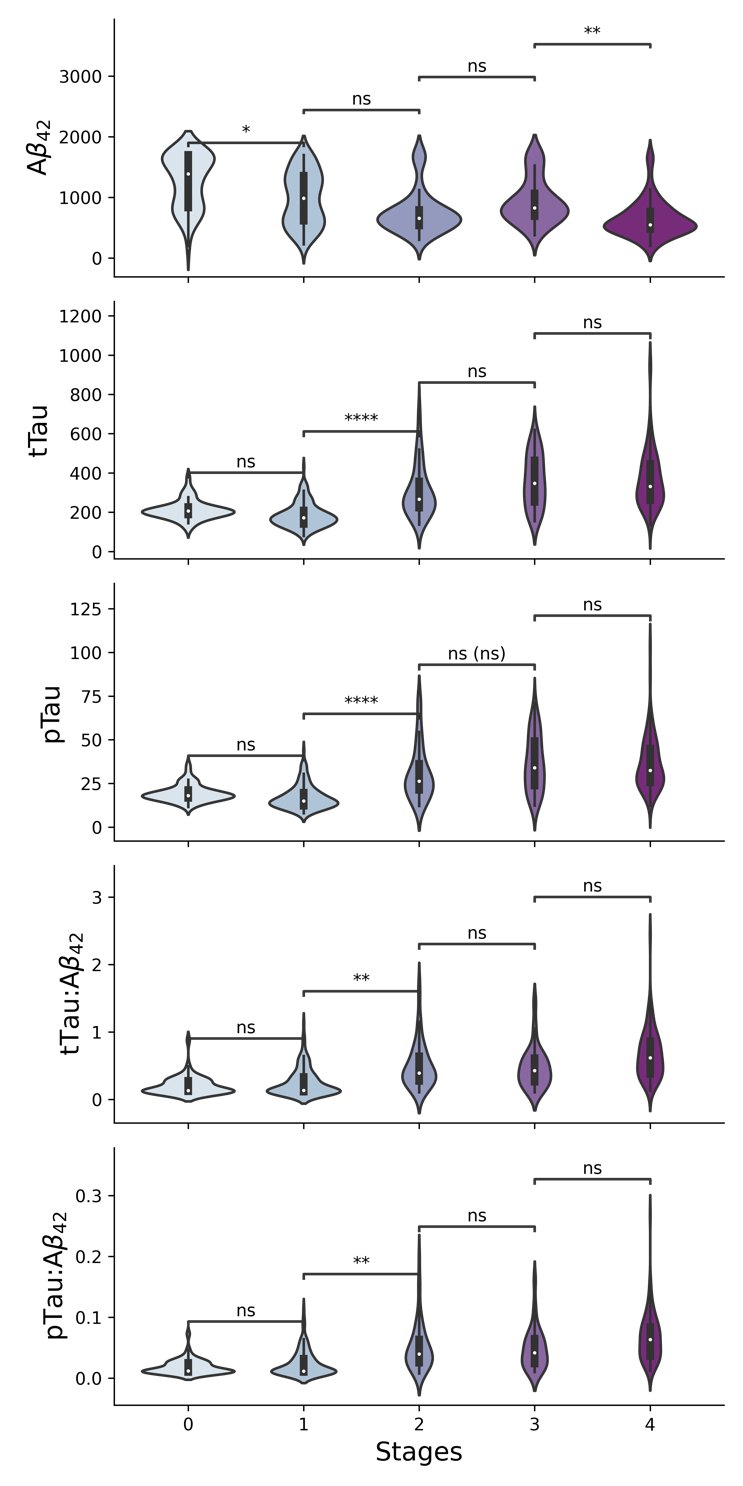
**

**Supplementary Figure S5**- The distribution for important disease pathology markers which were not used by the EBM model for learning the event sequence or staging individuals (n=392). The figure shows the distribution of these markers as a function of disease stage inferred by EBM, across all subjects in the EHBS cohort. Aβ_42_ shows the earliest changes, as compared to tTau, pTau, and their ratios with Aβ_42_. Tau related markers (pTau and tTau) show strongest changes between the 1^st^ and 2^nd^ stages. P-values computed using Mann-Whitney test and corrected using the Holm-Bonferroni method (* p < 10^-4^, ** p < 10^-6^, *** p < 10^-8^, **** p < 10^-10^). The measurement units for Aβ_42_, tTau, pTau are in pg/mL.
